# One Year in the Extreme Isolation of Antarctica—Is This Enough to Modulate an “Allergic” Sensitization?

**DOI:** 10.3390/biomedicines10020448

**Published:** 2022-02-15

**Authors:** Matthias Feuerecker, Claudia Strewe, Martina Aumayr, Tim Heitland, Ulrich Limper, Brian Crucian, Sarah Baatout, Alexander Choukér

**Affiliations:** 1 Department of Anesthesiology, University Hospital, LMU Munich, 81377 Munich, Germany; matthias.feuerecker@med.uni-muenchen.de (M.F.); claudia.strewe@med.uni-muenchen.de (C.S.); 2 MacroArray Diagnostics GmbH, Lemböckgasse 59/Top 4, A-1230 Vienna, Austria; aumayr@macroarraydx.com; 3 Alfred-Wegener-Institut, Helmholtz-Zentrum für Polar-und Meeresforschung, 27568 Bremerhaven, Germany; tim.heitland@awi.de; 4 Institut für Luft-und Raumfahrtmedizin, Deutsches Zentrum für Luft-und Raumfahrt (DLR), 51147 Cologne, Germany; Ulrich.Limper@dlr.de; 5 NASA-Johnson Space Center, Houston, TX 77058, USA; brian.crucian-1@nasa.gov; 6 Radiobiology Unit, Belgian Nuclear Research Centre (SCK CEN), 2400 Mol, Belgium; sarah.baatout@sckcen.be

**Keywords:** Antarctica, space mission like confinement, allergy, chip-based multiplex assay, sensitization, immune system

## Abstract

(1) Background: After spending a year wintering in Antarctica, individual expedition members have reported increased or even new allergic reactions to environmental allergens after their return. (2) Methods: Blood samples from five overwintering crews were analyzed using the chip based multiplex ALEX Allergy Explorer (MacroArray Diagnostics GmbH, Austria). (3) Results: About one third of the 39 participants displayed specific IgEs against pollen. In most individuals, kinetics showed a reduction in the specific IgE at the time about nine months after deployment to Antarctica. Five participants had the highest specific IgE levels after returning to the “normal” world. The examination of the specific IgE relative to house dust mites and storage mites showed different kinetics. Six out of 10 had the highest specific IgE concentrations at the inner Antarctic measurement time point. These data corresponded well to the general situation in the stations. At the stations themselves, there were almost no pollen particle load, especially at Concordia. (4) Conclusions: Antarctic long-term confinement can induce an altered immune function, which is in some individuals pronounced after return to the familiar allergen environment. Future prospective studies in larger cohorts are needed to further specify these first results.

## 1. Introduction

Antarctica is the most hostile continent on Earth—it is the coldest, driest, and windiest, but is also the cleanest place on our planet. The average annual temperature ranges from about −10 °C (coast) to −60 °C at the highest parts of inner Antarctica [1]. Only during summertime temperatures can rise to maximal +10 °C at the coastal side. At inland locations, they hardly ever rise above −30 °C.

About a decade ago, our group started immunological human studies in this extreme environment in one year overwintering crews, primarily focusing on innate and adaptive immune alterations [2,3] but also on general stress responses. These investigations took place at two Antarctic stations: one at the coastal side (the German Neumayer III Station) and one at a high plateau in inner Antarctica (the French–Italian Concordia Station). In contrast to the Neumayer III Station, where some wildlife can be found, Concordia Station is completely isolated from wildlife. The closest coast is about 1000 km away from Concordia Station. Due to its location at a high plateau (~3200 m) overwintering crews face a significant reduction in available oxygen partial pressure leading to various changes in the human body [4,5].

During the last years, several personal communications have reached us in which returnees from Antarctica reported that they experienced severe or even new allergy typical symptoms such as hay fever, wheezing, or even throat swelling. For example, one overwintering participant described that a new onset of asthmatic symptoms occurred in the presence of their neighbor’s cat after their return. Another one, who had previously had allergic reactions (rash, wheezing) to cats and dogs complained about newly developed hay fever, wheezing, and throat swelling in the presence of grass and flowers, also after return.

These communications brought our attention to retrospectively investigate potential sensitizations and type-1 hypersensitivity (“allergic”) typical symptoms in five previously studied overwintering crews at Neumayer III and Concordia Station.

## 2. Materials and Methods

### 2.1. Station Description and Original Study Protocol

Two different Antarctic research stations served as study locations for the original investigations [2,3,5]: Concordia, the French–Italian inner-continental station located at an altitude of 3233 m (pressure level ∼640 to 650 hPa) and Neumayer III, the German coastal station at Atka Bay in the northeast Weddell Sea on the Ekström shelf ice at sea level.

In contrast to the Northern Hemisphere, the seasons are inversed—the Antarctic summer lasts from November to February and the winter from May to August. Due to the close location to the South Pole, a light/dark cycle is missing during the Antarctic summer, resulting in 24 h of constant sunlight. Summertime average outside temperatures are around −40 °C at Concordia and −3 °C at Neumayer III. During wintertime, no sunlight is present for almost three months. This leads to outside temperatures around −60 °C and sometimes also to −80 °C at Concordia. At Neumayer III, winter darkness lasts some weeks less/fewer with average temperatures of −30 °C. These extreme temperatures are not the only challenges for the expeditioners. Particularly at Concordia, the humidity is very low, which causes a very dry environment, leading to mucosal defects—nose bleeding is hereby common during the first weeks of deployment. Both stations are completely isolated (no real evacuation possible) from the rest of the world from mid-February to mid-October. All telecommunication/internet is only possible via satellite and depends on the weather situation.

The original study CHOICE (Consequences of long-term **C**onfinement and **H**ypobaric Hyp**O**xia on **I**mmunity in the Antarctic **C**oncordia **E**nvironment) included sample collection at baseline (about two months before departure (pre)), on a monthly basis, and about 3–4 months after return (post). For this retrospective investigation, an “inner Antarctic” time point was chosen with the longest isolation time from the outer world—for both stations, this was the September collection (Sept). Samples of three overwintering crews from Concordia and samples of two crews from Neumayer III were analyzed. In detail, blood samples (serum tubes) from 23 Concordia crew members (21 males/two females) and 16 Neumayer participants were analyzed (11 males/five females). Complete sample sets were present in 34 cases, whereas five sets from Concordia were incomplete (2 × September and 3 × post missing).

### 2.2. Biochemical Measurements

As only small amounts of blood existed for further sensitization analyses, the study team decided to use the chip based multiplex ALEX Allergy Explorer (MacroArray Diagnostics GmbH, Vienna, Austria) containing more than 280 allergen extracts and molecular allergens.

ALEX tests were performed from 200 µL serum plasma at the MacroArray Diagnostics Laboratory at 1230 Vienna, Austria. This test measures the semi-quantitative total IgE concentrations as well as quantitative allergen specific IgE. The ALEX test clusters eight different allergen groups (pollen, mites, microorganisms, plant-based food, animal-derived food, insects and venoms, epithelial tissues of animals, others) and further investigated cross-reactive allergen families containing 12 different components (Supplementary Table S1).

General classification of total IgE (kU/L) concentrations in the ALEX analyses for adults are as follows:
<20 kU/L—Allergy unlikely20–100 kU/L—Allergy possible>100 kU/L—Allergy likely

### 2.3. Questionnaire

All participants were contacted via email during springtime 2020 and asked to fill in a questionnaire including questions about allergy typical reactions, history of allergy, and changes after return from Antarctica.

### 2.4. Statistical Analyses

All data were tested for normal distribution using the Shapiro–Wilk test. Between-group comparisons were performed using the Mann–Whitney Rank Sum test for non-parametric data and a *t*-test for normally distributed data. A *p*-value < 0.05 was regarded as statistically significant.

The majority of the data are presented in this report as raw data to provide the maximal possible scientific overview and not to “diminish” biological effects after statistical calculations.

Data in figures are displayed as single values. SigmaPlot^®^ (Systat, Software, Chicago, IL, USA) and IBM SPSS Statistics (V24, Armonk, NY, USA) were used for the statistical analyses and figure design.

## 3. Results

### 3.1. Demographic Data

Aside from the smaller group size (23 vs. 16), no significant statistical differences in demographics were present between the stations (Table 1).

### 3.2. The ALEX Test

#### Total IgE

**Figure 1:**
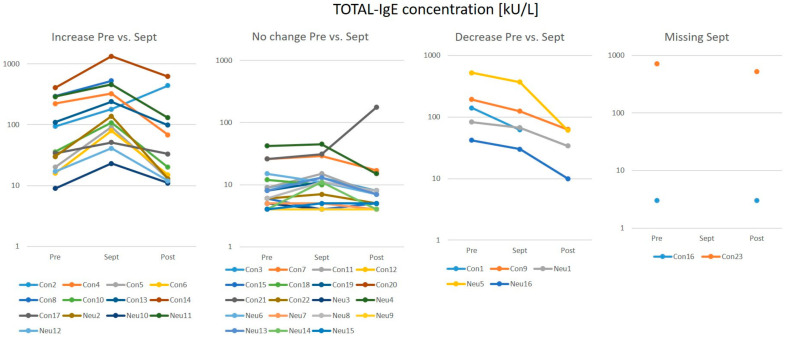
Total IgE concentrations in overwinterers separated in groups by the change in September.

Thirteen out of the 39 participants had at one of the three time points a total IgE (tIgE) level above 100 kU/L. According to the test definition, an allergy is likely when tIgE is >100 kU/L. When looking at the September tIgE concentrations, 13 participants showed elevated levels compared to the baseline (Figure 1).

Alterations of tIgE concentrations were defined here if values were affected by more than ±10 kU/L. Aside from two participants in which tIgE levels increased further, all levels dropped again after return.

### 3.3. Specific IgEs

By further analyzing the ALEX test, we observed in 26 participants elevated specific IgE (sIgE) levels according to the test ranges/classification (low, moderate, high, very high; Supplement Table S1).

Seven overwinterers had only one elevated sIgE level of a cluster and 19 multiple ones (Supplement Table S2). Of the seven participants, four showed moderate and three low sIgE levels. Only one showed a total IgE at one time point above 100kU/L. The majority of the participants with positive sIgE was classified in one cluster high (six) or very high (eight). The number one sensitization was toward pollen (17, high/very high 9), followed by food (15, high/very high 3), insects (12, high/very high 0), mites (10, high/very high 7), pets (7, high/very high 2), and fungal spores and yeast 3 (high/very high 2).

### 3.4. Grass Pollen

Individual analyses of the allergen extracts (AE) and the molecular allergens (MA) of the grass pollen cluster revealed, aside from 2 (Con4, Sec c pollen; Con6, Lol p1) out of 13, a reduction in specific IgE levels in September. September values were in most cases also lower than the post data. In two individuals (Con2, Con9), some AE and MA were moderately higher after overwintering when compared to the baseline values (Con2 Phl p, Phl p2, Con9 Lol p1).

**Figure 2:**
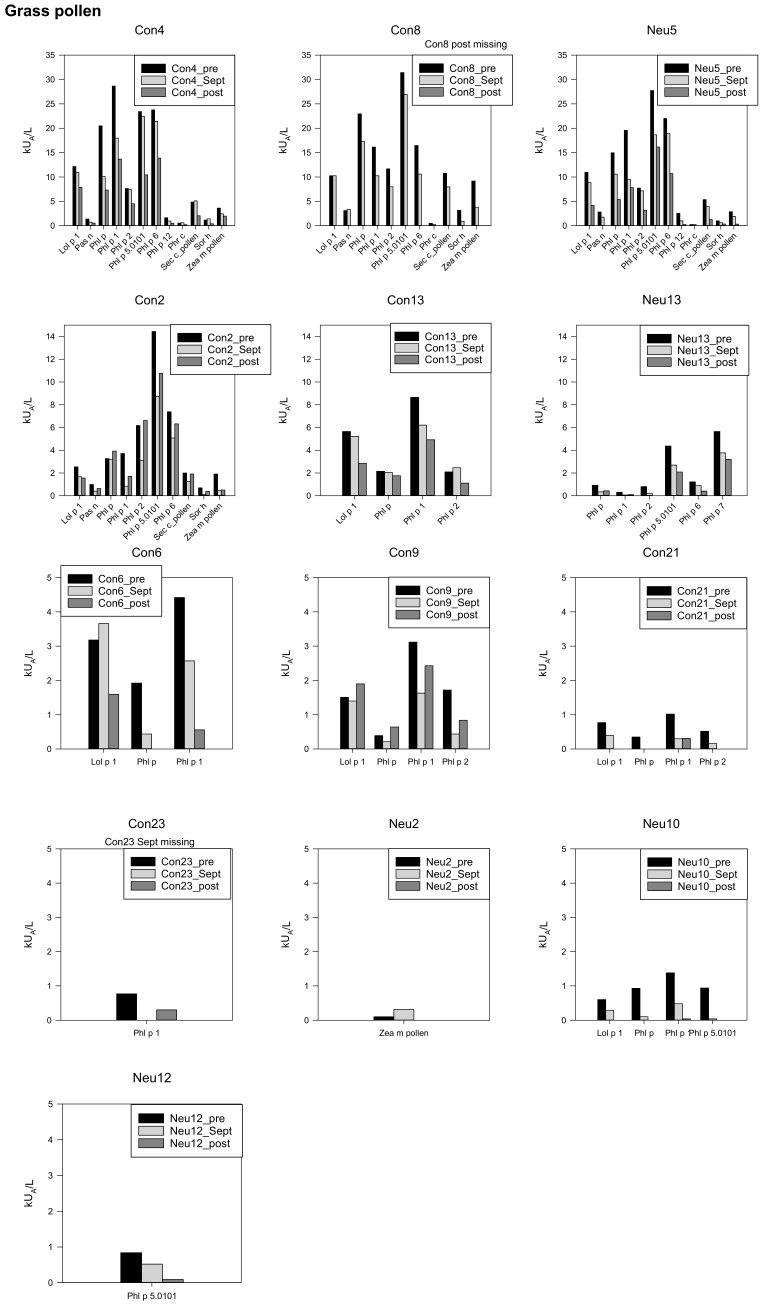
Specific IgEs against grass pollen, individual data, starting with the highest concentrations in the first line, *y*-axis adapted to the respective concentrations.

The specific IgE concentrations for grass pollen were classified at any time point very high for three participants, high in three, moderate (three), and low in four individuals (Figure 2).

### 3.5. Tree Pollen

**Figure 3:**
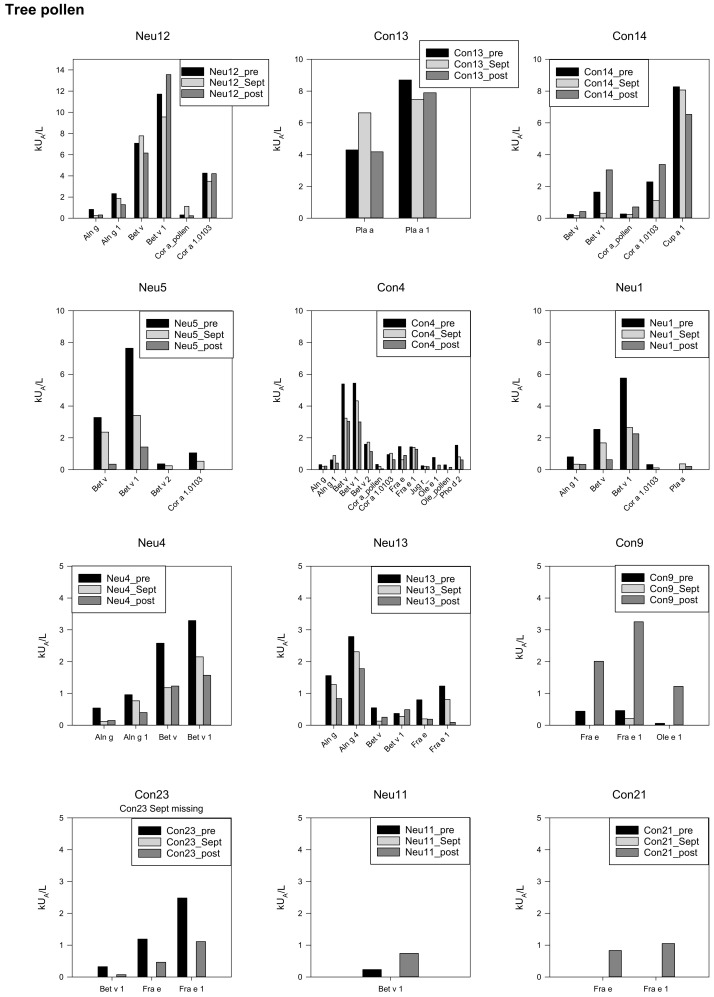
Specific IgEs against tree pollen, individual data, starting with the highest concentrations in the first line, *y*-axis adapted to the respective concentrations.

Twelve participants had sensitizations toward tree pollen. In general, concentrations were high in six cases, moderate in four cases, and low in two cases. Interestingly, three overwinterers showed higher sIgE levels in September than at the baseline (Neu12, Bet v; Con4 Aln g1, Bet v2, Con13 Pla a). Five individuals had the highest levels for some specific IgEs after the Antarctic expedition (Neu11, Neu12, Con9, Con14, Con21). Two of them (Neu11, Con21) were within the low range (0.3–1 kUA/L). Con21 had no specific IgE before Antarctica, but low levels afterward (Figure 3).

### 3.6. House Dust Mites & Storage Mites

**Figure 4:**
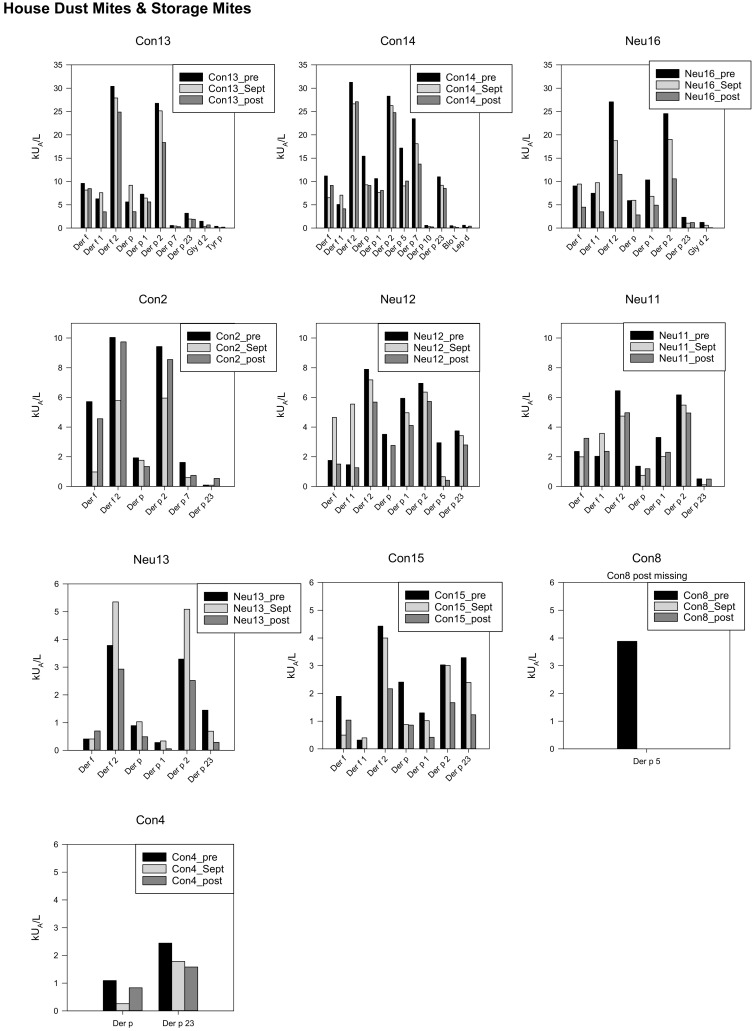
Specific IgEs against house dust mites and storage mites, individual data, starting with the highest concentrations in the first line, *y*-axis adapted to the respective concentrations.

Ten participants showed specific IgEs against AE and MA toward house dust mites and storage mites. Three had very high, four high, and three moderate sIgE concentrations. Six individuals had peak levels for some AE/MA in September (Con13, Con14, Neu11, Neu12, Neu13, Neu16). The majority of the post values were lower than the pre data (Figure 4).

### 3.7. Microorganisms

**Figure 5:**
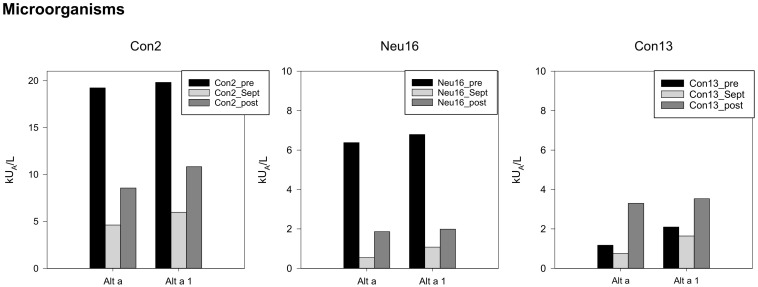
Specific IgEs against house dust mites and storage mites, individual data, starting with the highest concentrations on the left side, *y*-axis adapted to the respective concentrations.

Three participants were sensitized toward Alternaria alternans. One had very high, one high, and one had moderate levels. In September, the levels showed the lowest values. One participant presented the highest levels after the expedition, whereas the other two had remarkably lower ones compared to the baseline (Figure 5).

### 3.8. Pets

**Figure 6:**
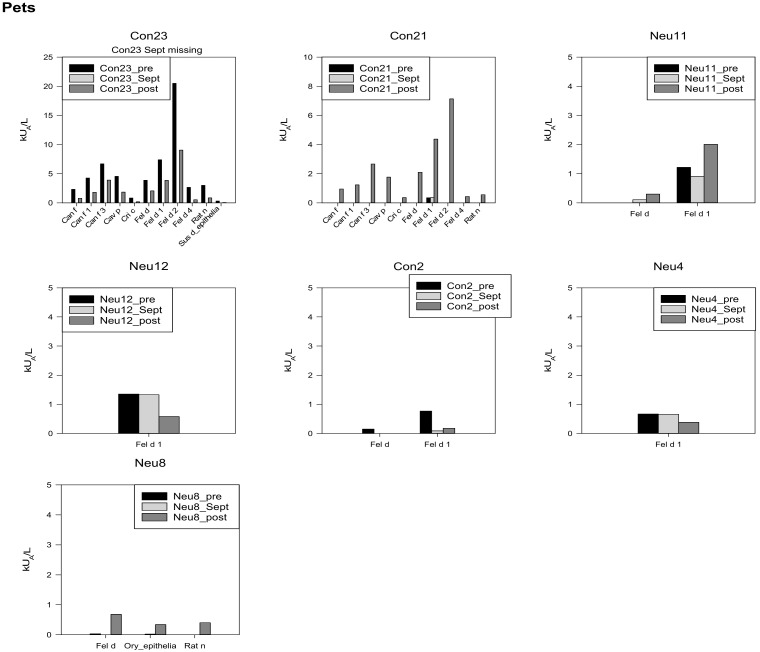
Specific IgEs against pets, individual data, starting with the highest concentrations in the upper left corner, *y*-axis adapted to the respective concentrations.

Specific IgE reactions toward pet AE and MA were observed in seven participants. One with very high levels, one with high, two with moderate, and three with low levels. Interestingly, three showed the highest levels after return. One participant developed a new sensitization toward various pet AE and ME (Con21) (Figure 6).

### 3.9. Questionnaires and ALEX Results

The return rate of the questionnaire was low (11 out of 39 (28.2%)). Five declared that they had no history of allergy or any typical sensitization, whereas six indicated a history of allergy including hay fever, wheezing (asthmatic reactions), and increased swelling after insect bites.

Two of the participants with a history of allergy experienced more hay fever after return (Con4, Con15). Total IgE levels after return were in both cases lower than pre departure. Specific IgEs showed no increase in the post data collection, respectively.

Two other expeditioners reported on the newly developed hay fever after their return (Con21, Con23). In one case (Con21), total IgE concentration was the highest at the post time point (176 vs. 26 (pre) kU/L). A new sensitization after return against the ash extract and molecular allergen could be detected. In the other case (Con23), no alterations in terms of an increase were observed. Total IgE levels dropped from pre 719 to post 521 kU/L.

Another subject (Con9) who had hay fever from olive trees explained that this reaction was gone after its return. Total IgE levels in this case were also the lowest after return, but the specific IgE toward ash (Fra e, Fra e1), olive (Ole e1), and the Ole e1 family were higher post expedition. This is in contrast to the clinical description of an improvement in allergic symptoms (Table 2) and speaks to the limited association between elevated tIgE and clinical symptoms.

## 4. Discussion

As initially described, this retrospective investigation was initiated after the incidence of personal communications increased where returnees wintering over for a year in Antarctic stations experienced more allergic reactions than they had at home before deployment to Antarctica. To the best of the authors’ knowledge, this is the second report 44 years after Lund´s et al. [6] first investigation into the effects of prolonged isolation in Antarctica and the modulation of the allergic state.

Due to the few available sample volumes from five overwintering crews, the study team tried to obtain the maximal possible scientific outcome one could achieve on a retrospective basis. In this light, we have chosen a newer chip based multiplex assay (ALEX Allergy Explorer) as it contained a very broad variety of allergen extracts and molecular allergens. The ALEX test showed comparable results to the Immuno Solid-phase Allergen Chip (ISAC, Thermo Fisher Scientific, Waltham, MA, USA) test [7], whereas the ISAC test was able to investigate only 112 different single molecules from 48 different allergen sources.

In addition to the specific IgEs, the ALEX test also measures the total IgE concentrations. In contrast to previous findings from Lund [6] on total IgE concentrations, which showed only a variation around the baseline values in 21 subjects, we were able to detect in 33% of the subjects an increase in total IgE levels during the Antarctic stay (September). The increase was hereby defined if values changed by more than ±10 kU/L. As demonstrated in an earlier investigation at Concordia [2], the environmental conditions/isolation caused an upregulated global immune response. This immune response was especially evident in a specific T-cell stimulation assay imitating the original delayed type hypersensitivity skin test (Merieux^®^, Chicago, IL, USA) with the evaluation of its type IV reaction [8]. The altered total IgE levels might be a further indicator that in some individuals, this environmental setup alters general immune functions, also on a type 1 reaction. Of course, the majority (19 participants) had no tIgE changes at the September time point. When looking into the literature, one can find that even in severe asthmatics, total serum IgEs show limited within-patient variability [9]. However, one also has to mention that severe asthma patients have many medications, especially systemic steroids and some monoclonal antibodies against various cytokines that could blunt any change in total IgE concentrations. It is also known that total IgE can be elevated by psychological stress and that due to its half-life of around 60 h, a variety of non-allergic influences could have impacted the here presented levels. Additionally, no age correction was performed in this investigation, possibly affecting the results as allergen-specific and total IgE levels decrease with age [9,10]. In general, one has to take into account that normal total IgE levels can also range from 2 to 214 units/mL. This was shown in a 2014 published manuscript with 1376 healthy children and 128 adults in the United States [11]. Certainly, one cannot over interpret the here presented findings. Nevertheless, they might be an interesting observation caused by the special Antarctic environment and living conditions.

From the specific IgE levels of the pollen cluster, one can assess that in the majority of the participants, sIgEs levels were lower in September and also after return. Interestingly, five individuals had the highest levels after return toward distinct tree pollen allergen extracts and molecular allergens. Certainly, elevated total IgE and specific IgE concentrations should not be stressed as singular markers in the diagnosis of allergies or allergic reactions. However, these findings in the overwintering/returning crew could give a hint that somehow sensitization to pollen could be affected by the year-long isolation from such allergens and lead to the personal communications presented in the introduction.

Due to the long distance to the next Antarctic coast (~1000 km) with wildlife possibly transporting pollen, one can assume that there is no significant pollen present at Concordia Station. Though Neumayer III is situated at the coast on shelf-ice, the closest island (Bouvet Island) with some spare vegetation is more than 1600 km away. Significant amounts of pollen are not expected to be on this station. Overall, the here presented data on pollen sIgEs are in good accordance with the general known sIgE kinetics that specific IgE tends to decrease when not exposed and go up in response to exposure [12].

Elevated sIgEs after return were also present in two other clusters—microorganisms (one out of three) and pets (three out of seven). The kinetic over time was otherwise similar to the pollen cluster.

In terms of pet allergens, one has to acknowledge that there is also no animal life at Concordia Station. At Neumayer III, winter breeding places of the Emperor penguin can be found not far from the station. The ALEX test does not investigate bird allergens, which could have detected possible new sensitizations. Most sensitizations were in context with cat allergens. After several months, specific IgE levels such as the Fel d1 (cat allergen) are, in general, shed [12,13]. At the inner Antarctic time point (Sept), none of the sIgE investigating pet sensitizations was elevated. This is confirmative as there is no typical pet allergen exposition at the stations.

Somehow different was the cluster “house dust mites and storage mites”. Six out of 10 individuals presented the highest peak sIgE levels in September. One explanation therefore can be that these stations are not at all allergen free areas and trigger specific IgE generation. Though the humidity is low at these stations and additionally, a low oxygen pressure is present at Concordia, van Houdt et al. [14] described the airborne bacterial population at Concordia. The predominant microflora at the station was associated with human activity and as a consequence, influence the surface contamination. These factors could have led to the observed increase in house dust and storage mites in September.

In contrast to Neumayer Station, wildlife does not exist at the inner-Antarctic Concordia Station, possibly leading to a lower overall environmental antigen load and altered immune stimulation normally occurring in a regular environment [15,16,17]. Though the station groups were not big in number, one could see a difference in the unspecific immune reactivity by tIgE levels, as nine of the Concordia participants and only four from Neumayer showed an increase in September.

As Hamilton et al. [18] stated that total IgE and specific IgE concentrations as single markers are not sufficient to diagnose an allergy, we tried to retrospectively obtain information on the history of allergy. Six participants (from 11 returned questionnaires) indicated a history of allergy before the expedition to Antarctica. The description of the reactions and the measured tIgE and sIgE did not correlate well. The main reason for this might be that the time point of the post blood draw (3–4 months after return) and the late questionnaire survey are too divergent.

### Limitations

This investigation and the generated data do have several shortcomings as to the retrospective data acquisition and the divergent questionnaire/sampling time points. This must be carefully differentiated between the presence of allergen specific IgE, a single lab parameter, from a clinical allergic sensitization. Since up to 30% of individuals can have allergen-specific IgE via serum test and/or skin test and have no clinical symptoms, most allergists would be unwilling to call someone “allergic” because their total IgE was elevated. The ALEX test cluster of total IgE uses this common definition allergy likely/unlikely depending on total IgE. In this report, this separation was not used. Instead, the authors tried to show new findings on sensitizations in an extraordinary environment.

Further limitations have to be addressed when looking into the data of specific IgE levels. Potential sensitizations with pollen (after unpacking stored food packages during winter over) or after sweeping distinct areas could have influenced the measurements in September as the supplies were not packed in an allergen-free environment. In future studies, one certainly has to measure the allergen load (e.g., out of dust samples (Der P1 or Der F1)). Food sensitizations detected by the ALEX test were not further analyzed and discussed as food sensitizations/allergies are considered as different entities.

Taken together, the here presented data provide additional information on a possible altered immune function in the sense of a type 1 reaction during the Antarctic deployment, which is in some individuals pronounced after return to the familiar allergen environment.

A further study in a prospective designed model is warranted—if such a risk of new sensitization and “overshooting” reaction exists—as such findings could have an impact on future long-term expeditions or space missions (e.g., to the Moon and Mars) when an allergic sensitization during mission could have potentially more serious consequences when maximal medical treatment is not guaranteed. That the here presented results are not random display other observations made in extreme conditions of living environments such as on the International Space Station published by NASA from one of the authors’ team (B. Crucian) [19,20,21]. These manuscripts describe changes in the first line of immune defense, the microbiome and relate this to stress events, indicating the role of the environmental challenge on the one hand (so being confined and stressed), but will also allow us to bridge these phenomena to other immune dysregulations as reported (e.g., viral reactivations). The here presented data and the possibly underlying immunological mechanisms add another element to this understanding of complex immune responses, though the exact causes of these sensitizations can only be answered in a speculative way. As initially described, this special Antarctic environment leads to significant changes in distinct immunological pathways [2]. To date, alterations of immunological pathways at Antarctica cannot be directly linked to allergic sensitizations.

## Figures and Tables

**Table 1:** Demographic data of the overwintering crews (height and body weight from one Concordia crew missing, female), data are mean ± SD (range).

	Concordia	Neumayer III	All
Number	23	16	39
Gender [m/f]	21/2	11/5	32/7
Age during WO [years]	36.5 ± 10.9 (23–62)	33.3 ± 4.8 (25–42)	35.2 ± 9.0 (23–62)
Height [cm]	175.3 ± 7.1 (165–191)	175.6 ± 9.0 (157–188)	175.5 ±7.8 (157–191)
Weight [kg]	76.6 ± 13.2 (58–107)	78.3 ± 17.0 (52–109)	77.3 ± 14.7 (52–109)
Body mass index [kg/m^2^]	24.9 ± 3.8 (19.7–33.3)	25.1 ± 3.8 (19.8–34.4)	25.0 ± 3.8 (19.7–34.4)

**Table 2:** Overview of the ALEX test summary of all participants who returned the questionnaire, color explanation: no color/rose (0) = <0.3 kUA/L (negative or uncertain), yellow (1) = 0.3–1 kUA/L (low IgE level), orange (2) = 1–5 kUA/L (moderate IgE level), red (3) = 5–15 kUA/L(high IgE level), dark red (4) = >15 kUA/L (Very high IgE level). No color and rose lines show different subjects. Grey columns show separations of test clusters.

Participant.	Pollen			Mites	Micro-Organisms	Plant-Based Food				Animal-Derived Food		Insects & Venoms	Epithelial Tissues of Animals	Others		Cross-Reactive Allergen Families									Total IgE		History of Allergy	Type of Allergy, Symptoms and Development
	Grass Pollen	Tree Pollen	Weed	House Dust Mites & Storage Mites	Fungal Spores & Yeast	Legumes	Fruits	Vegetables & mushrooms	Nuts & Seeds	Fish & Seafood	Meat	Bee, Wasp	Pets	Latex		Profilin	PR-10	Ole e 1 Family	LTPs	Lipocalins	NPC2	Serum albumin	Tropo-myosin		(kU/L)			
Con2_pre	3	0	1	3	4	0	0	0	0	0	0	0	1	0		0	0	1	0	0	3	0	0		94		no	
Con2_Sept	3	0	0	3	3	0	0	0	0	0	0	0	0	0		0	0	0	0	0	3	0	0		179			
Con2_post	3	0	0	3	3	0	0	0	0	0	0	0	0	0		0	0	0	0	0	3	0	0		441			
Con3_pre	0	0	0	0	0	0	0	0	0	0	0	0	0	0		0	0	0	0	0	0	0	0		9		no	
Con3_Sept	0	0	0	0	0	0	0	0	0	0	0	1	0	0		0	0	0	0	0	0	0	0		13			
Con3_post	0	0	0	0	0	0	0	0	0	0	0	0	0	0		0	0	0	0	0	0	0	0		8			
Con4_pre	4	3	0	2	0	1	0	1	1	0	0	2	0	2		2	3	2	0	0	0	0	0		223		yes	hay fever, reactions to insect bites, younger age allergy towards cats/dogs, food reactions
Con4_Sept	4	2	0	2	0	1	0	1	1	0	0	1	0	0		2	2	2	0	0	0	0	0		324			
Con4_post	3	2	0	2	0	1	1	0	1	0	0	1	0	2		2	2	2	0	0	0	0	0		68			afterwards, same reactions as before, but more hay fever
Con6_pre	2	0	0	0	0	0	0	0	0	0	0	0	0	0		0	0	0	0	0	0	0	0		16		no	
Con6_Sept	2	0	0	0	0	0	0	0	0	0	0	0	0	0		0	0	0	0	0	0	0	0		80			
Con6_post	2	0	0	0	0	0	0	0	0	0	0	0	0	0		0	0	0	0	0	0	0	0		15			
Con9_pre	2	1	1	0	0	0	0	0	0	0	0	0	0	0		0	0	1	0	0	0	0	0		193		yes	hay fever (pollen from olive trees)
Con9_Sept	2	0	1	0	0	0	0	0	0	0	0	0	0	0		0	0	0	0	0	0	0	0		124			
Con9_post	2	2	0	0	0	0	0	0	0	0	0	0	0	0		0	0	2	0	0	0	0	0		63			no hay fever after return
Con15_pre	0	0	0	2	0	0	0	0	0	0	0	0	0	0		0	0	0	0	0	2	0	0		6		yes	hay fever (varying with years), dust mites
Con15_Sept	0	0	0	2	0	0	0	0	0	0	0	0	0	0		0	0	0	0	0	2	0	0		4			
Con15_post	0	0	0	2	0	0	0	0	0	0	0	0	0	0		0	0	0	0	0	2	0	0		5			worse hay fever after return
Con17_pre	0	0	0	0	0	0	0	0	0	0	0	1	0	0		0	0	0	0	0	0	0	0		34		no	
Con17_Sept	0	0	0	0	0	0	0	0	0	0	0	0	0	0		0	0	0	0	0	0	0	0		51			
Con17_post	0	0	0	0	0	0	0	0	0	0	0	0	0	0		0	0	0	0	0	0	0	0		33			
Con21_pre	2	0	0	0	0	0	0	0	0	0	0	0	1	0		0	0	0	0	0	0	0	0		26		yes afterwards	
Con21_Sept	1	0	0	0	0	0	0	0	0	0	0	0	1	0		0	0	0	0	0	0	0	0		31			
Con21_post	1	2	0	0	0	0	0	0	0	0	1	0	3	0		0	0	2	0	2	0	3	0		176			newly experienced hay fever directly after return for a couple of days
Con23_pre	1	2	2	0	0	0	0	0	0	0	2	0	4	0		0	1	2	0	2	0	4	0		719		yes	cats, dogs, rats -> wheezing
Con23_Sept	missing																											
Con23_post	1	2	0	0	0	0	0	0	0	0	0	0	3	0		0	0	2	0	2	0	3	0		521			new allergic reactions to gras/flowers ->hay fever, wheezing, throat swelling
Neu4_pre	0	2	0	0	0	0	0	0	1	0	0	0	1	0		0	2	0	0	0	0	0	0		41		yes	grass and rabbit allergy
Neu4_Sept	0	2	0	0	0	0	0	0	0	0	0	0	1	0		0	2	0	0	0	0	0	0		45			
Neu4_post	0	2	0	0	0	0	0	0	0	0	0	0	1	0		0	2	0	0	0	0	0	0		15			
Neu6_pre	0	0	0	0	0	0	0	0	0	2	0	0	0	0		0	0	0	0	0	0	0	0		15		no	
Neu6_Sept	0	0	0	0	0	0	1	0	0	2	0	1	0	0		0	0	0	0	0	0	0	0		11			
Neu6_post	0	0	0	0	0	0	0	0	0	2	0	0	0	0		0	0	0	0	0	0	0	0		7			

## Data Availability

The data presented in this study are available on request from the corresponding author. The data are not publicly available due to ethical reasons.

## Supplementary Materials

The following are available online at https://www.mdpi.com/article/10.3390/biomedicines10020448/s1. Table S1: Example of an ALEX test result summary; Table S2: Specific IgEs in all overwinterers: Due to no specific IgEs, the following categories were deleted to increase clarity: grain, spices, milk, egg, cockroach, animals, ficus, and hops, CCD; Deleted items for cross-reactive allergen families: polcalcin, storage proteins, parvalbumin, CCD.
