# Supplementary material for: One Year in the Extreme Isolation of Antarctica—Is This Enough to Modulate an “Allergic” Sensitization?
Source: Biomedicines. 2022 Feb 15;10(2):448. doi: 10.3390/biomedicines10020448 (PMC8962425; doi:10.3390/biomedicines10020448)
Supplement: Supplementary file 1 [file biomedicines-10-00448-s001.zip › biomedicines-1583418-supplementary.pdf]

Table S1. Example of an ALEX test result summary.

**Pollen**	Grass Pollen	3	**Cross-Reactive Allergen Families**	
	Tree Pollen	2	Polcalcin	0
	Weed Pollen	0	Profilin	2
**Mites**	House Dust Mites and Storage Mites	2	PR-10	2
**Microorganisms**	Fungal Spores and Yeast	0	Ole e 1 Family	2
**Plant-Based Food**	Legumes	1	LTPs	0
	Grain	0	Storage Proteins	0
	Spices	0	Lipocalins	0
	Fruits	1	NPC2	0
	Vegetables and mushrooms	0	Serum albumin	0
	Nuts and Seeds	1	Parvalbumin	0
**Animal-Derived Food**	Milk	0	Tropomyosin	0
	Egg	0	CCD	0
	Fish and Seafood	0		
	Meat	0	**Total IgE (kU/L)**	**68**
**Insects and Venoms**	Bee, Wasp	0		
	Cockroach	0		
**Epithelial Tissues of Animals**	Pets	0		
	Animals	0		
**Others**	Latex	2		
	Ficus an Hops	0		
	CCD	0		

**Highest measured IgE concentration per allergen group**				
**0**	**1**	**2**	**3**	**4**
<0.3 kUA/L	0.3–1 kUA/L	1–5 kUA/L	5–15 kUA/L	>15 kUA/L
Negative or uncertain	Low IgE level	Moderate IgE level	High IgE level	Very high IgE level

Table S2. *Specific IgEs in all overwinterers:* Due to no specific IgEs, the following categories were deleted to increase clarity: grain, spices, milk, egg, cockroach, animals, ficus, and hops, CCD; Deleted items for cross-reactive allergen families: polcalcin, storage proteins, parvalbumin, CCD. color explanation: no color/rose (0) = <0.3 kUA/L (negative or uncertain), yellow (1) = 0.3–1 kUA/L (low IgE level), orange (2) = 1–5 kUA/L (moderate IgE level), red (3) = 5–15 kUA/L(high IgE level), dark red (4) = >15 kUA/L (Very high IgE level). No color and rose lines show different subjects. Grey columns show separations of test clusters.

Participant	Pollen			Mites	Microorganisms	Plant-Based Food				Animal-Derived food		Insects & Venoms	Epithelial Tissues of Animals	Others	Cross-reactive Allergen Families								Total IgE	History of Allergy	Type of Allergy, symptoms and development
	Grass Pollen	Tree Pollen	Weed	House Dust Mites & Storage Mites	Fungal Spores & Yeast	Legumes	Fruits	Vegetables & mushrooms	Nuts & Seeds	Fish & Seafood	Meat	Bee, Wasp	Pets	Latex	Profilin	PR-10	Ole e 1 Family	LTPs	Lipocalins	NPC2	Serum albumin	Tropomyosin	(kU/L)		
Con2_pre	3	0	1	3	4	0	0	0	0	0	0	0	1	0	0	0	1	0	0	3	0	0	94	no	
Con2_Sept	3	0	0	3	3	0	0	0	0	0	0	0	0	0	0	0	0	0	0	3	0	0	179		
Con2_post	3	0	0	3	3	0	0	0	0	0	0	0	0	0	0	0	0	0	0	3	0	0	441		
Con3_pre	0	0	0	0	0	0	0	0	0	0	0	0	0	0	0	0	0	0	0	0	0	0	9	no	
Con3_Sept	0	0	0	0	0	0	0	0	0	0	0	1	0	0	0	0	0	0	0	0	0	0	13		
Con3_post	0	0	0	0	0	0	0	0	0	0	0	0	0	0	0	0	0	0	0	0	0	0	8		
Con4_pre	4	3	0	2	0	1	0	1	1	0	0	2	0	2	2	3	2	0	0	0	0	0	223	yes	hay fever, reactions to insect bites, younger age allergy towards cats/dogs, food reactions
Con4_Sept	4	2	0	2	0	1	0	1	1	0	0	1	0	0	2	2	2	0	0	0	0	0	324		
Con4_post	3	2	0	2	0	1	1	0	1	0	0	1	0	2	2	2	2	0	0	0	0	0	68		afterwards, same reactions as before, but more hayfever
Con5_pre	0	0	0	0	0	0	0	0	0	1	0	1	0	0	0	0	0	0	0	0	0	0	20	unknown	
Con5_Sept	0	0	0	0	0	0	0	0	0	2	0	1	0	0	0	0	0	0	0	0	0	0	92		
Con5_post	0	0	0	0	0	0	0	0	0	1	0	0	0	0	0	0	0	0	0	0	0	0	13		
Con6_pre	2	0	0	0	0	0	0	0	0	0	0	0	0	0	0	0	0	0	0	0	0	0	16	no	
Con6_Sept	2	0	0	0	0	0	0	0	0	0	0	0	0	0	0	0	0	0	0	0	0	0	80		
Con6_post	2	0	0	0	0	0	0	0	0	0	0	0	0	0	0	0	0	0	0	0	0	0	15		
Con8_pre	4	0	2	2	0	0	0	0	0	0	0	1	0	0	0	0	0	0	0	0	0	0	294	unknown	
Con8_Sept	4	0	2	0	0	0	0	0	0	0	0	2	0	0	0	0	0	0	0	0	0	0	525		
Con8_post	missing																						missing		

Con9_pre	2	1	1	0	0	0	0	0	0	0	0	0	0	0		0	0	1	0	0	0	0	0		193		yes	hay fever (pollen from olive trees)
Con9_Sept	2	0	1	0	0	0	0	0	0	0	0	0	0	0		0	0	0	0	0	0	0	0		124			
Con9_post	2	2	0	0	0	0	0	0	0	0	0	0	0	0		0	0	2	0	0	0	0	0		63			no hay fever after return
Con10_pre	0	0	0	0	0	0	0	0	0	0	1	0	0	0		0	0	0	0	0	0	0	0		36		unknown	
Con10_Sept	0	0	0	0	0	0	0	0	0	0	0	0	0	0		0	0	0	0	0	0	0	0		108			
Con10_post	0	0	0	0	0	0	0	0	0	0	0	0	0	0		0	0	0	0	0	0	0	0		20			
Con13_pre	3	3	2	4	2	3	3	1	2	0	1	0	0	0		0	0	0	3	0	4	0	0		110		unknown	
Con13_Sept	3	3	1	4	2	3	3	1	1	0	0	0	0	0		0	0	0	3	0	4	0	0		240			
Con13_post	2	3	2	4	2	3	3	1	1	0	0	0	0	0		0	0	0	3	0	4	0	0		99			
Con14_pre	0	3	0	4	0	0	0	0	0	1	0	2	0	0		0	2	0	0	0	4	0	1		405		unknown	
Con14_Sept	0	3	0	4	0	0	0	0	0	1	0	1	0	0		0	2	0	0	0	4	0	1		1335			
Con14_post	0	3	0	4	0	0	1	0	0	0	1	2	0	0		0	2	0	0	0	4	0	0		618			
Con15_pre	0	0	0	2	0	0	0	0	0	0	0	0	0	0		0	0	0	0	0	2	0	0		6		yes	hay fever (varying with years), dust mites
Con15_Sept	0	0	0	2	0	0	0	0	0	0	0	0	0	0		0	0	0	0	0	2	0	0		4			
Con15_post	0	0	0	2	0	0	0	0	0	0	0	0	0	0		0	0	0	0	0	2	0	0		5			worse hay fever after return
Con17_pre	0	0	0	0	0	0	0	0	0	0	0	1	0	0		0	0	0	0	0	0	0	0		34		no	
Con17_Sept	0	0	0	0	0	0	0	0	0	0	0	0	0	0		0	0	0	0	0	0	0	0		51			
Con17_post	0	0	0	0	0	0	0	0	0	0	0	0	0	0		0	0	0	0	0	0	0	0		33			
Con21_pre	2	0	0	0	0	0	0	0	0	0	0	0	1	0		0	0	0	0	0	0	0	0		26		yes afterwards	
Con21_Sept	1	0	0	0	0	0	0	0	0	0	0	0	1	0		0	0	0	0	0	0	0	0		31			
Con21_post	1	2	0	0	0	0	0	0	0	0	1	0	3	0		0	0	2	0	2	0	3	0		176			newly experienced hay fever directly after return for a couple of days
Con23_pre	1	2	2	0	0	0	0	0	0	0	2	0	4	0		0	1	2	0	2	0	4	0		719		yes	cats, dogs, rats -> wheezing
Con23_Sept	missing																								missing			
Con23_post	1	2	0	0	0	0	0	0	0	0	0	0	3	0		0	0	2	0	2	0	3	0		521			new allergic reactions to gras/flowers ->hay fever, wheezing, throat swelling
Neu1_pre	0	3	1	0	0	0	2	0	1	0	0	2	0	0		0	3	0	2	0	0	0	0		83		unknown	

Neu1_Sept	0	2	0	0	0	0	2	0	1	0	0	2	0	0		0	2	0	2	0	0	0	0		67			
Neu1_post	0	2	0	0	0	0	1	0	1	0	0	1	0	0		0	2	0	1	0	0	0	0		34			
Neu2_pre	0	0	0	0	0	0	0	0	0	0	0	1	0	0		0	0	0	0	0	0	0	0		30		unknown	
Neu2_Sept	1	0	0	0	0	0	0	0	0	0	0	2	0	0		0	0	0	0	0	0	0	0		138			
Neu2_post	0	0	0	0	0	0	0	0	0	0	0	1	0	0		0	0	0	0	0	0	0	0		13			
Neu3_pre	0	0	0	0	0	0	0	0	0	0	0	2	0	0		0	0	0	0	0	0	0	0		5		unknown	
Neu3_Sept	0	0	0	0	0	0	0	0	0	0	0	1	0	0		0	0	0	0	0	0	0	0		4			
Neu3_post	0	0	0	0	0	0	0	0	0	0	0	0	0	0		0	0	0	0	0	0	0	0		4			
Neu4_pre	0	2	0	0	0	0	0	0	1	0	0	0	1	0		0	2	0	0	0	0	0	0		41		yes	grass and rabbit allergy
Neu4_Sept	0	2	0	0	0	0	0	0	0	0	0	0	1	0		0	2	0	0	0	0	0	0		45			
Neu4_post	0	2	0	0	0	0	0	0	0	0	0	0	1	0		0	2	0	0	0	0	0	0		15			
Neu5_pre	4	3	0	0	0	0	0	0	1	0	0	0	0	0		2	3	0	0	0	0	0	0		523		unknown	
Neu5_Sept	4	2	0	0	0	0	1	0	0	0	0	1	0	0		1	2	0	0	0	0	0	0		368			
Neu5_post	4	2	0	0	0	0	0	0	0	0	0	1	0	0		0	2	0	0	0	0	0	0		61			
Neu6_pre	0	0	0	0	0	0	0	0	0	2	0	0	0	0		0	0	0	0	0	0	0	0		15		no	
Neu6_Sept	0	0	0	0	0	0	1	0	0	2	0	1	0	0		0	0	0	0	0	0	0	0		11			
Neu6_post	0	0	0	0	0	0	0	0	0	2	0	0	0	0		0	0	0	0	0	0	0	0		7			
Neu8_pre	0	0	0	0	0	0	0	0	0	2	0	0	0	0		0	0	0	0	0	0	0	0		6		unknown	
Neu8_Sept	0	0	0	0	0	0	0	0	0	1	0	0	0	0		0	0	0	0	0	0	0	0		11			
Neu8_post	0	0	0	0	0	0	0	0	0	3	0	0	1	0		0	0	0	0	0	0	0	0		8			
Neu10_pre	2	0	0	0	0	0	0	0	0	0	0	0	0	0		0	0	0	0	0	0	0	0		9		unknown	
Neu10_Sept	1	0	0	0	0	0	0	0	0	0	0	0	0	0		0	0	0	0	0	0	0	0		23			
Neu10_post	0	0	0	0	0	0	0	0	0	0	0	0	0	0		0	0	0	0	0	0	0	0		11			
Neu11_pre	0	0	0	3	0	0	0	0	0	2	0	1	2	0		0	0	0	0	0	3	0	0		290		unknown	
Neu11_Sept	0	0	0	3	0	0	0	0	0	0	0	0	1	0		0	0	0	0	0	3	0	0		458			
Neu11_post	0	1	0	2	0	0	0	0	0	1	0	0	2	0		0	1	0	0	0	2	0	0		130			

Neu12_pre	1	3	0	3	0	2	0	1	0	0	0	0	2	0		0	3	0	0	0	3	0	0		17		unknown	
Neu12_Sept	1	3	0	3	0	2	0	0	0	0	0	0	2	0		0	3	0	0	0	3	0	0		41			
Neu12_post	0	3	0	3	0	3	0	1	0	0	0	0	1	0		0	3	0	0	0	3	0	0		12			
Neu13_pre	3	2	0	2	0	0	0	0	0	0	0	0	0	0		0	1	2	0	0	2	0	0		8		unknown	
Neu13_Sept	2	2	0	3	0	0	0	0	0	0	0	0	0	0		0	0	1	0	0	3	0	0		13			
Neu13_post	2	2	0	2	0	0	0	0	0	0	0	0	0	0		0	1	0	0	0	2	0	0		7			
Neu16_pre	0	0	0	4	3	0	0	0	0	0	0	0	0	0		0	0	0	0	0	4	0	0		42		unknown	
Neu16_Sept	0	0	0	4	2	0	0	0	0	2	0	0	0	0		0	0	0	0	0	4	0	0		30			
Neu16_post	0	0	0	3	2	0	0	0	0	0	0	0	0	0		0	0	0	0	0	3	0	0		10			
